# Hazardous heat exposure among incarcerated people in the United States

**DOI:** 10.1038/s41893-024-01293-y

**Published:** 2024-03-05

**Authors:** Cascade Tuholske, Victoria D. Lynch, Raenita Spriggs, Yoonjung Ahn, Colin Raymond, Anne E. Nigra, Robbie M. Parks

**Affiliations:** 1 Department of Earth Sciences, Montana State University, Bozeman, MT, USA.; 2 GeoSpatial Core Facility, Montana State University, Bozeman, MT, USA.; 3 Department of Environmental Health Sciences, Mailman School of Public Health, Columbia University, New York, NY, USA.; 4 Department of Geography and Atmospheric Science, University of Kansas, Lawrence, KS, USA.; 5 Joint Institute for Regional Earth System Science and Engineering, University of California, Los Angeles, CA, USA.

## Abstract

Climate change is predicted to increase the frequency of potentially hazardous heat conditions across the United States, putting the incarcerated population of 2 million at risk for heat-related health conditions. We evaluate the exposure to potentially hazardous heat for 4,078 continental US carceral facilities during 1982–2020. Results show that the number of hot days per year increased during 1982–2020 for 1,739 carceral facilities, primarily located in the southern United States. State-run carceral facilities in Texas and Florida accounted for 52% of total exposure, despite holding 12% of all incarcerated people. This highlights the urgency for enhanced infrastructure, health system interventions and treatment of incarcerated people, especially under climate change.

## Reporting summary

Further information on research design is available in the Nature Portfolio Reporting Summary linked to this article.

Incarcerated people in the United States are at high risk for heat-related morbidity and mortality due to their physical confinement, social isolation and high rates of chronic mental and physical illnesses^1–3^. Unlike the large majority of the US population, who have access to air conditioning (central and any air conditioning equipment)^4^—the most effective individual-level intervention to mitigate heat exposure^1^—many of the 2 million incarcerated people^5^ are in the 44 states that do not universally provide air conditioning in carceral facilities^6,7^.

Identifying where incarcerated people are exposed to hazardous heat conditions is fundamental to advancing environmental justice for one of the most marginalized and disempowered communities in the United States^3^. Yet researchers and policymakers have largely overlooked how heat impacts incarcerated people^3,8,9^, in part due to perceptions that their physical suffering is justified^3^. As climate change accelerates, the United States will experience more frequent, intense and longer heatwaves that may disproportionately affect incarcerated people^8^.

Here we evaluate recent exposure to and the trends of potentially hazardous heat conditions during 1982–2020 for all 4,078 operational and populated carceral facilities (referring to prisons, jails, immigration detention facilities and other carceral facilities) in the continental United States (Methods). We define potentially hazardous heat as the number of days per year where the indoor maximum wet bulb globe temperature (WBGT_max_) exceeds 28 °C, the threshold defined by the US National Institute for Occupational Safety and Health (for acclimated populations to limit humid heat exposure under moderate workloads (234–349 W)^10^.

**Fig. 1 |:**
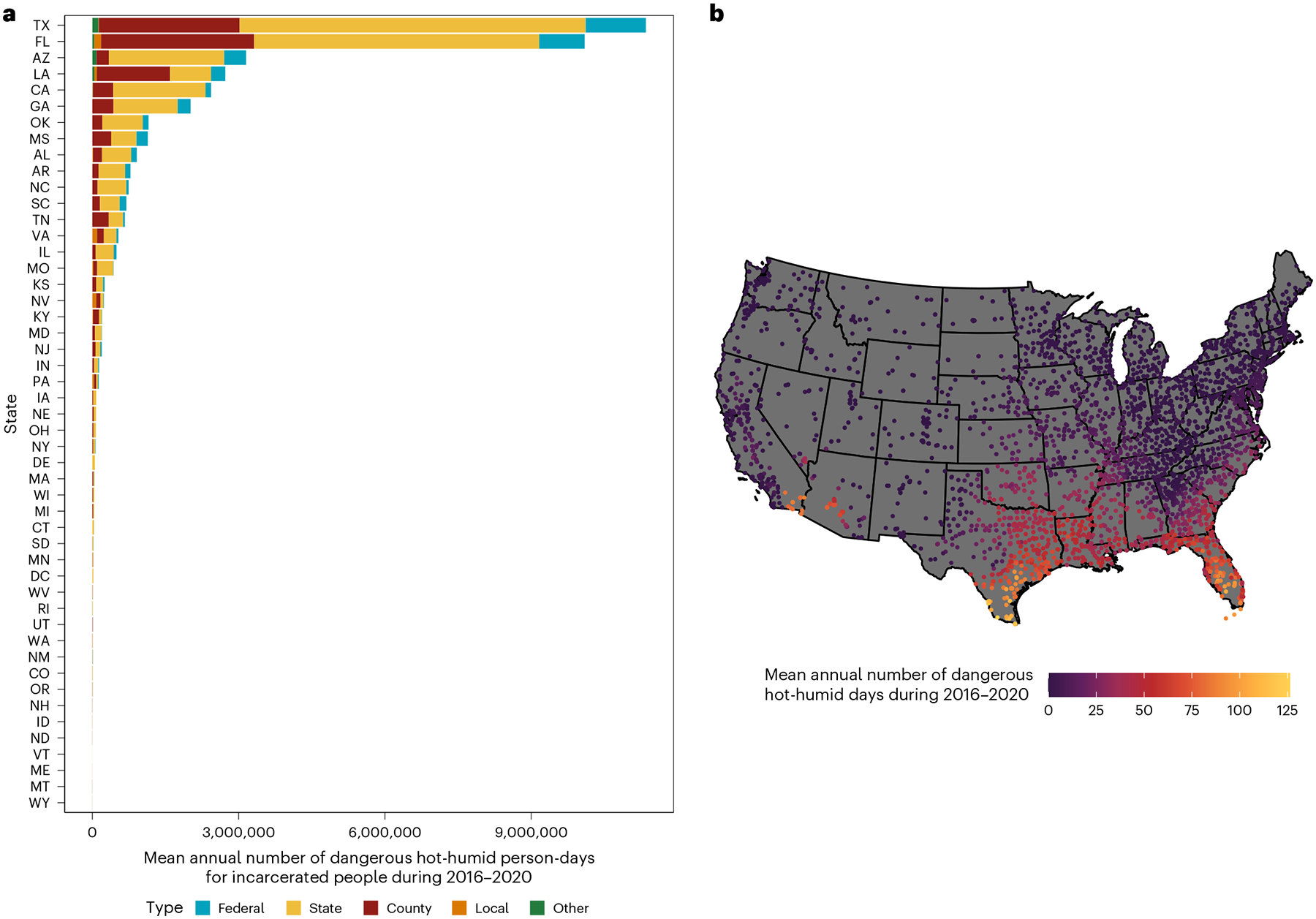
Mean annual exposure during 2016–2020 to potentially hazardous heat in carceral facilities within the continental United States. **a**,**b**, For each carceral facility (*N* = 4,078), metrics of potentially hazardous heat measured by: the number of person-days WBGT_max_ exceeded 28 °C for incarcerated people by state and carceral facility type (**a**) and the number of days WBGT_max_ exceeded 28 °C for each carceral facility (**b**). State names are abbreviated with standard two-letter abbreviations.

During 2016–2020, there were, on average, 41.3 million person-days of heat exposure annually at carceral facilities in the United States. State prisons accounted for 61% (24.5 million person-days) of total exposure (Fig. 1a), followed by county jails (11.1 million person-days; 27%). The estimated 145,240 people in Texas and 98,941 in Florida housed in state-run carceral facilities in 2018—12% of all incarcerated people in the United States—accounted for 52% of total exposure (Fig. 1a). One hundred eighteen carceral facilities, largely in southern California, Arizona, Texas and inland Florida, experienced on average 75 days or more per year where WBGT_max_ exceeded 28 °C (Fig. 1b). Air conditioning in carceral facilities in these states is spotty or relies on a less effective cooling system such as evaporative cooling^11^ where air conditioning even exists^6,7^. Across all US carceral facilities, the Starr County Jail, a county facility in Rio Grande, Texas, which held 249 people in 2018, experienced the largest number of day per year WBGT_max_ exceeded 28 °C on average during 2016–2020 (126.2 days per year). We include additional analyses by further carceral facility types in Supplementary Figs. 1 and 2.

**Fig. 2 |:**
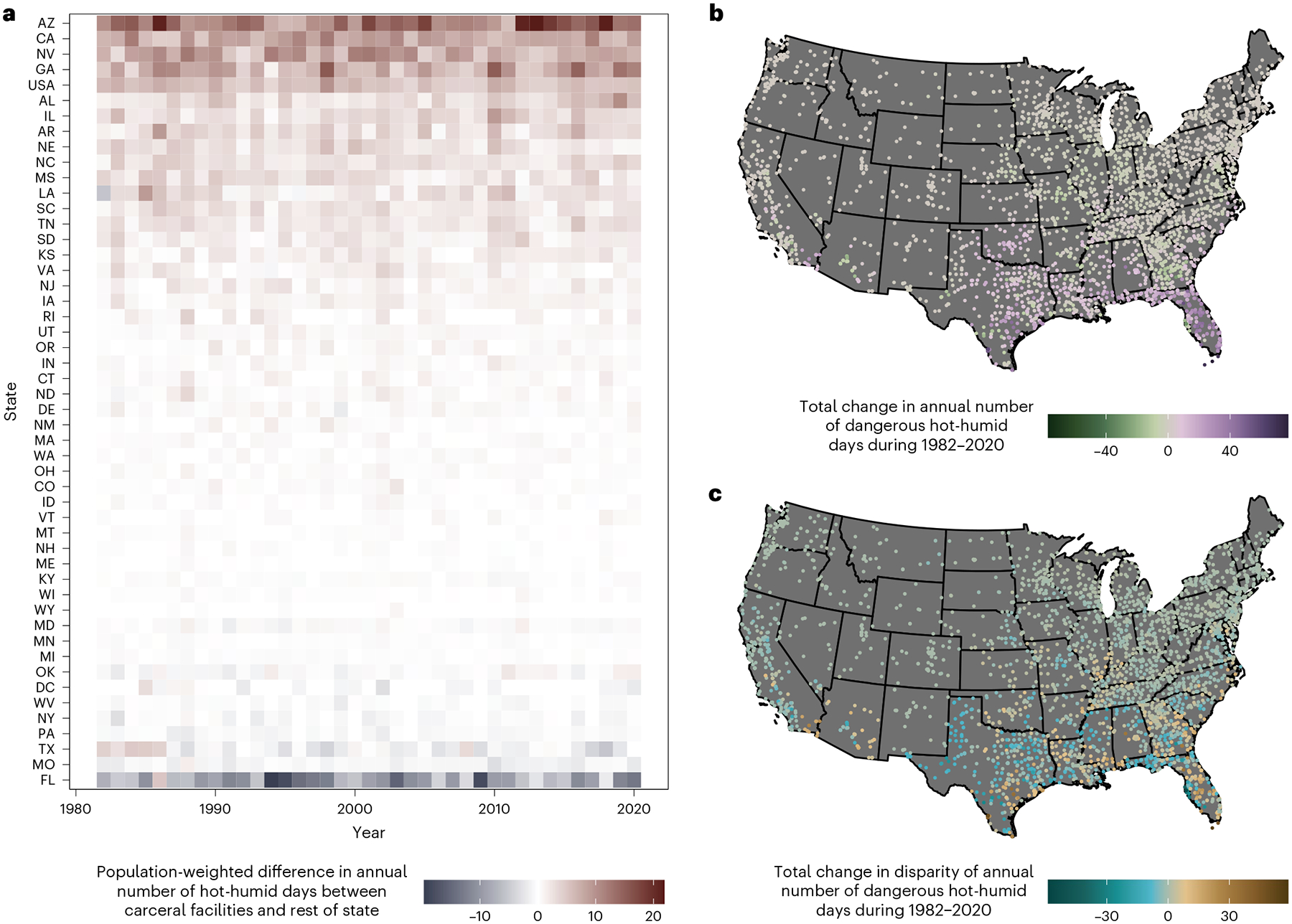
Trends in annual exposure during 1982–2020 to potentially hazardous heat in carceral facilities within the continental United States. **a**, Populationweighted difference between the annual number of days WBGT_max_ exceeded 28 °C at the location of carceral facilities versus all other locations in the continental United States during 1982–2020, overall and stratified by state, ordered by average population-weighted difference. **b**,**c**, The total change in the number of days WBGT_max_ exceeded 28 °C per year for each carceral facility in the continental United States during 1982–2020 (**b**) and the total change in disparity in number of days WBGT_max_ exceeded 28 °C per year for each carceral facility in the continental United States, compared with the rest of the state the carceral facility is located, during 1982–2020 (**c**).

During 1982–2020, carceral facility locations were, on average, exposed to 5.5 more days per year where WBGT_max_ exceeded 28 °C annually compared to locations without carceral facilities (Fig. 2a). However, there was a considerable amount of variation by year, with a maximal disparity of 9.8 more days at carceral facilities than locations without carceral facilities in 1998 and a minimal disparity of 3.5 days in 1994. Arizona, California and Nevada ranked as the top three states with the greatest exposure disparities (Fig. 2a). Carceral facilities in Arizona experienced 13.1 more days per year than the rest of the state and 40.9 more days compared to the entire continental United States during 1982–2020 on average. Statistics comparing the characteristics of incarcerated and non-incarcerated people are found in Supplementary Tables 1 and 2.

In 2018, 915,627 people in the United States, 45% of the estimated total incarcerated population, were housed in 1,739 carceral facilities with an annual increase in the number of days per year WBGT_max_ exceeded 28 °C during 1982–2020 (Fig. 2b). These facilities are primarily located in the southern United States, which faced the greatest number of potentially hazardous heat days per year since 1982 (Fig. 2b). Carceral facilities in Florida experienced on average 22.1 more days in 2020 compared to 1982, the greatest increase in humid heat days for all continental states, consistent with previous work finding that the largest relative increases in heat stress are expected at latitudes closer to the equator^12^. The greatest overall increase relative to the state was for Webb County Jail, Texas, with 58.7 more days than the rest of Texas in 2020 compared with 1982 (Fig. 2c). We also present results from Figs. 1 and 2 with alternative thresholds of 26 °C and 30 °C (Supplementary Figs. 3–6).

The majority of carceral facilities in the southern United States have experienced a rapid increase in potentially hazardous heat exposure since the 1980s and are located in states that do not have mandatory conditioning access for state-run institutions^6,7^. Whereas physically this rapid increase in heat exposure is a result of anthropogenic climate change, land-cover and land-use change, including an urban heat island effect caused by the materials used to construct carceral facilities^3^, this geographic disparity also reflects state-level criminal justice policies, as southern states have the highest imprisonment rates in the United States (though not necessarily highest jailing rates)^13^ and the inherent differential effects of climate change. Throughout the country, including in the Northeast and Midwest, many locations with carceral facilities also experienced an increasing number of days WBGT exceeded 28 °C compared with other locations. This continuing intensification limits the effectiveness of heat-mitigation plans (if they exist at all) at non-air-conditioned facilities^11^.

That we found carceral facilities are systematically exposed to an increasing number of potentially hazardous heat days compared with other areas of the United States is plausible for several reasons. First, carceral facilities are often built where there is availability of low-cost land and limited resistance of local communities^14^. In many states, areas that meet these criteria are in sparsely populated desert or swampy environments^5^. Zoning laws in urban environments and security issues also favour construction in isolated, desert-like areas^14^. The lack of disparity we identify in Florida is an exception probably due to the North–South climate gradient, with a relative dearth of carceral facilities in the most hot-humid, but economically wealthy and densely populated, southern tip. We found that the top four most-exposed states to potentially hazardous heat days per year were Texas, Florida, Arizona and Louisiana, all of which do not provide universal air conditioning to their prisons^7^, potentially creating a double burden of increased exposure and vulnerability.

Incarcerated people have few options to reduce the impact of hazardous heat^3,7,9^, and these marginalized communities are often disproportionately susceptible to the effect of heat exposure given pre-existing health conditions. An estimated 43% of the state prison population has a previous mental health diagnosis^15^, and people on psychotropic medications are at increased risk for heat illness^16^. Exposure to elevated heat can also cause both acute health effects, such as heat stroke or mortality, and long-term damage. For example, chronic dehydration strains kidney function and those with chronic heat exposure have been shown to have higher rates of kidney disease^17^. Such vulnerabilities are especially relevant given restrictive prison policies with respect to drinking water and other potential heat-adaptation tools^3^.

Though there have been recent declines, the incarcerated population of the United States has increased by 500% over the past four decades^18^. People of colour are overrepresented in carceral facilities and compose an estimated two-thirds of the total incarcerated population. The prison population is also ageing, with one in seven serving life in prison^19^, potentially resulting in greater overall heat vulnerability to those incarcerated. Structural racism manifests in persistently higher proportions and rates of incarcerated people being people of colour^20^. Acknowledging and accounting for the role structural racism plays in incarceration is critical to understand both key vulnerabilities to heat and contextualizing solutions to heat exposure. Appropriate preparation for periods of elevated heat is also critical. For example, seasonal forecasts could help facilities prepare for summer heatwaves to reduce the impacts of hazardous conditions for incarcerated communities.

Our work highlights how incarcerated populations in the United States are systematically exposed to potentially hazardous heat with the greatest exposure and rates of increase concentrated in state-run institutions. Federal, state and local laws mandating safe temperature ranges, enhanced social and physical infrastructure and health system interventions could mitigate the effect of hazardous heat. Underlying this is the need for a fundamental overhaul to the perception and treatment of incarcerated people in environmental public health policy and regulatory action. Further work is critical to comprehensively characterize the vulnerability of the United States incarcerated population to heat and how heat impacts health, to build reliable and validated datasets of cooling mechanisms in prisons and jails, to directly measure indoor temperatures in prisons and jails and to deploy adaptation measures to mitigate the worst impacts of climate-related stressors. Doing so is critical to environmental justice, particularly for incarcerated people with limited social and political agency.

## Methods

We assigned daily WBGT_max_ estimates to 4,078 carceral facility locations for the United States during 1982–2020. WBGT_max_ is constructed from high-resolution (4 km) daily maximum 2 m air temperatures (*T*_max_) and maximum vapour pressure deficit (VPD_max_) from the Parameter-elevation Regressions on Independent Slopes Model (PRISM) dataset^21^. *T*_max_ and VPD_max_ are used to construct daily maximum heat index (HI_max_) following the US National Weather Service’s procedure^22^, which is converted to indoor, or shaded, WBGT_max_ using a quadratic transform that assumes fixed wind speeds (0.5 m s^−1^) and no radiated heat (daily WBGT_max_ estimates in Supplementary Information). Facility location and population data are from Homeland Infrastructure Foundation-Level Data (HIFLD), produced by the Department of Homeland Security^5^. We evaluated PRISM-derived WBGT_max_ against ECMWF Reanalysis v5 (ERA5)- and Hadley Centre Integrated Surface Database (HadISD)-derived WBGT_max_ in Supplementary Figs. 7 and 8.

We then define potentially hazardous heat frequency as the number of days per year where the maximum WBGT_max_ exceeded 28 °C, the threshold used by the US National Institute for Occupational Safety and Health for acclimated populations to limit heat exposure under moderate workloads (234–349 W)^10^, and it is used widely in environmental epidemiological research^23,24^. Exposure during 2016–2020 is measured by multiplying the number of incarcerated people housed at each carceral facility in 2018 by the average number of days WBGT_max_ exceeded 28 °C per year during 2016–2020. Annual disparity between incarcerated and locations without carceral facilities is measured by taking the population-weighted difference between the number of days WBGT_max_ exceeded 28 °C at the location of a facility and the rest of the state. Population weighting fairly reflects the experience of a population to heat stress. To measure the annual rate of change in annual heat exposure, we fit linear regressions to the count of days WBGT_max_ exceeded 28 °C per year for each facility. A more detailed explanation of methods is in ‘Calculating humid heat exposure and trajectories of change metrics’ in Supplementary Information.

## Supplementary Material

Supplementary Information

**Supplementary information** The online version contains supplementary material available at https://doi.org/10.1038/s41893-024-01293-y.

## Data Availability

Data used for this analysis are available via https://github.com/sparklabnyc/temperature_prisons_united_states_2024. The data used in this study were created from the following datasets. Daily 4-km PRISM data during 1982–2020 and HIFLD data are freely available at https://prism.oregonstate.edu/recent/ and https://hifld-geoplatform.opendata.arcgis.com, respectively. National Center for Health Statistics bridged-race dataset (Vintage 2020) is available during 1990–2020 (https://www.cdc.gov/nchs/nvss/bridged_race.htm) and from the US Census Bureau before 1990 (https://www.census.gov/data/tables/time-series/demo/popest/1980s-county.html). Source data are provided with this paper.

## Code availability

All code to reproduce this work and underlying daily WBGT_max_ for each carceral facility during 1982–2020 and analytical products used here are freely available at https://github.com/sparklabnyc/temperature_prisons_united_states_2024.
