# Supplementary material for: Hazardous heat exposure among incarcerated people in the United States
Source: Nat Sustain. Author manuscript; Available in PMC 2026 Feb 21. (PMC12922630; doi:10.1038/s41893-024-01293-y)
Supplement: Supplementary Information [file NIHMS2146674-supplement-Supplementary_Information.pdf]

nature sustainability

Brief Communication

https://doi.org/10.1038/s41893-024-01293-y

Hazardous heat exposure among incarcerated people in the United States

In the format provided by the authors and unedited

Table of contents

List of Supplementary Tables

List of Supplementary Figures

Background

Objectives

Incarceration data

Weather and climate data

Daily $WBGT_{max}$ estimates

Secondary analyses

Validation of $WBGT_{max}$

Calculating humid heat exposure and trajectories of change metrics

Incarcerated vs. non-incarcerated statistics

References

List of Supplementary Tables

Supplementary Table 1. Race, sex, and some SES variables for state incarcerated populations compared to U.S. general population.

Supplementary Table 2. Race of incarcerated population compared to state population for CA, TX, AZ.

List of Supplementary Figures

Supplementary Figure 1. Mean annual exposure during 2016 - 2020 to potentially hazardous heat in Federal carceral facilities within the continental United States (N = 232), measured by the number of person-days WBGTmax exceeded 28°C for incarcerated people by state and Federal facility type (Administrative, Federal Correctional Institution (FCI), Immigration and Customs Enforcement (ICE), Private, United States Penitentiary (USP), and Work camps).

Supplementary Figure 2. The population-weighted difference between the annual number of days with heat stress conditions (defined as WBGTmax exceeding 28°C) at the location of Federal carceral facilities (Administrative, Federal Correctional Institution (FCI), Immigration and Customs Enforcement (ICE), Private, United States Penitentiary (USP), and Work camps) versus all other locations in the continental United States from 1982 - 2020 stratified by state.

Supplementary Figure 3. Mean annual exposure during 2016 - 2020 to potentially hazardous heat in carceral facilities within the continental United States (N=4,078), measured by: (a) the number of person-days $WBGT_{max}$ exceeded 26°C for incarcerated people by state and carceral facility type; and (b) the number of days $WBGT_{max}$ exceeded 26°C for each carceral facility.

Supplementary Figure 4. Mean annual exposure during 2016 - 2020 to potentially hazardous heat in carceral facilities within the continental United States (N=4,078), measured by: (a) the number of person-days $WBGT_{max}$ exceeded 30°C for incarcerated people by state and carceral facility type; and (b) the number of days $WBGT_{max}$ exceeded 30°C for each carceral facility.

Supplementary Figure 5. (a) Population-weighted difference between the annual number of days $WBGT_{max}$ exceeded 26°C at the location of carceral facilities versus all other locations in the continental United States during 1982 – 2020, overall and stratified by state, ordered by average population-weighted difference, (b) the total change in the number of number of days $WBGT_{max}$ exceeded 26°C per year for each carceral facility in the continental United States during 1982 – 2020, and (c) the total change in disparity in number of number of days $WBGT_{max}$ exceeded 26°C per year for each carceral facility in the continental United States, compared with the rest of the state the carceral facility is located, during 1982 – 2020.

Supplementary Figure 6. (a) Population-weighted difference between the annual number of days $WBGT_{max}$ exceeded 30°C at the location of carceral facilities versus all other locations in the continental United States during 1982 – 2020, overall and stratified by state, ordered by average population-weighted difference, (b) the total change in the number of number of days $WBGT_{max}$ exceeded 30°C per year for each carceral facility in the continental United States during 1982 – 2020, and (c) the total change in disparity in number of number of days $WBGT_{max}$ exceeded 30°C per year for each carceral facility in the continental United States, compared with the rest of the state the carceral facility is located, during 1982 – 2020.

Supplementary Figure 7. Monthly correlation of daily T_{max}, RH_{min}, and $WBGT_{max}$ during 2015 – 2020 for HadISD stations, PRISM climate grids, and ERA5 reanalysis data for the United States.

Supplementary Figure 8. Monthly correlation of daily T_{max}, RH_{min}, and $WBGT_{max}$ during 2015 – 2020 for HadISD stations, PRISM climate grids, and ERA5 reanalysis data for the United States for days HADISD stations reported T_{max} greater than 26°C.

Background

While previous work has assessed how heat impacts incarcerated people in the United States,[1] there is a critical need to quantify potentially hazardous heat conditions at carceral facilities.[2-4] Without this knowledge, the impact of more frequent periods of elevated heat[2] on incarcerated people cannot be contextualized nor framed against future climate projections. Identifying where incarcerated people may face especially increasingly high or regular exposure is essential to guide targeted interventions to reduce harm to incarcerated peoples' health.[4] Mapping the spatial and temporal pattern of potentially hazardous heat trajectories among incarcerated communities – as well as disparities in patterns and trends in exposure – can inform policy discussions to make necessary changes at the local, state, and federal levels.[2-4]

While data measuring indoor temperatures in prisons and jails in the United States is scarce, a study from Denmark found temperatures in prison cells to be 4-5°C above those outdoors in summer.[5] This is approximately what was also found in the Harlem Heat Project in New York City, a study of indoor domestic temperatures in domestic settings in Harlem, which is an area of New York with many residents who suffer cooling hardship.[6]

Objectives

Our objectives are to (1) characterize recent heat exposure at each carceral facility location and by facility type and state; (2) measure how heat exposure at carceral facility locations compares with the rest of the population nationally and by state; and (3) calculate the changes in the number of days per year $WBGT_{max}$ exceeded 28°C at carceral facilities since the 1980s. For objectives (1) and (2), we focus on recent years (2016 – 2020) because we are interested in the current heat exposure. For objective (3), we focus on the entire 1982 – 2020 period because we are interested in long-term trends. The underlying, carceral facility-level daily $WBGT_{max}$ records during 1982 - 2020 and the derived data used in our analysis are publicly available (Data and Code Availability).

Incarceration data

We used carceral facility (referring to prisons, jails, and other carceral facilities) locational boundaries (polygon latitudinal and latitudinal coordinates) and population data from the Homeland Infrastructure Foundation-Level Data (HIFLD), produced by the United States Department of Homeland Security.[7] We included 4,078 operational and populated prisons, jails, and carceral facilities including ICE detention centers, juvenile or geriatric facilities, and halfway houses in the continental United States in our analysis. Of these, there were 232 federal, 1,606 state, 2,142 county, and 73 local facilities. Twenty-five (0.6% of total) carceral facilities did not fall into these categories and were classed as 'other'. Texas had the single most prisons and jails (411 or 10.1% of total). In total, in 2018, there were 2,032,647 incarcerated people in included prisons and jails, of which 187,847 were in federal, 1,202,930 in state, 604,699 in county, 25,267 in local, and 11,904 in other. Texas was also the state with the single most incarcerated people (233,601 or 11.5% of total). The single largest prison by population was Cook County Jails, IL, with 8,216 incarcerated people.

Weather and climate data

The Parameter-elevation Relationships on Independent Slopes Model (PRISM) dataset from Oregon State University provides high-resolution (4 km grids) daily T_{max} and maximum vapor pressure deficit (VPD_{max}) from 1981 - to near present.[8] As described in[9-11], mean fields are produced by interpolating data from a dense network of weather stations with a spatial-weight regression model that uses landscape features like elevation and aspect to predict daily meteorological conditions across the continental United States (CONUS). PRISM data has

been well-validated[9-11] and shown to be well-suited for heat-related epidemiological research in the United States.[11] The 4-km dataset is freely available online from the PRISM Climate Group.

Daily $WBGT_{max}$ estimates

WBGT is a heat stress metric widely used in environmental epidemiology to assess associations between heat and human health across a range of contexts.[12,13] WBGT accounts for the non-linear interactions between air temperature, humidity, air speeds, and solar radiation.[14] But given that incarcerated people spend the vast majority of their time indoors and thus solar radiation is negligible, here we estimate indoor, or shaded, $WBGT_{max}$.[14] Exposure is defined as the number of days per year that $WBGT_{max}$ exceeded 28°C multiplied by the total estimated incarcerated population exposed (person-days per year).[13]

Daily T_{max} and VPD_{max} mean fields from PRISM were converted to approximated indoor or shaded $WBGT_{max}$ following the procedure used in previous work.[14,15] Following [11], first, VPD_{max} are converted to daily minimum relative humidity fields (eq. 1)

$$RH_{min} = \frac{\left[610.94\,Pa \cdot e^{\left(\frac{17.625 \cdot T_{max}}{243.04^{\circ}C + T_{max}}\right)}\right] - VPD_{max}}{610.94\,Pa \cdot e^{\left(\frac{17.625 \cdot T_{max}}{243.04^{\circ}C + T_{max}}\right)}} \text{ (eq. 1)}$$

with T_{max} in °C, VPD_{max} in Pa, and RH_{min} in %. Next, we combine T_{max}, converted to °F, and RH_{min} to create daily maximum heat index (HI_{max}) mean fields following the U.S. National Weather Service's (NWS) procedure.[16] To calculate HI_{max} for each day, we use T_{max} and RH_{min} to best align relatively humidity at the time the daily maximum temperature occurs during a given diurnal cycle.[9] NWS first estimates HI_{max} using the average between T_{max} and Steadman's simplified equation:

$$HI_{max} = 0.5(T_{max} + 0.5\,(T_{max} + 61.0 + (1.2(T_{max} - 68.0)) + 0.094RH_{min})) \text{ (eq. 2)}$$

If the resulting HI_{max} is greater than 80°F, then the complete Rothfusz equation is estimated as

$$HI_{max} - 42.379 + 2.049015237T_{max} - 42.379 + 2.04901523T_{max} - .00683783T_{max}^2 - .05481717RH_{min}^2 + .00122874T_{max}^2RH_{min} + .00085282T_{max}^2\,RH_{min}^2 - .00000199T_{max}^2RH_{min}^2 \text{ (eq. 3)}$$

with the following adjustments: if T_{max} between 80°F – 112°F and RH_{min} less than 13%, adjustment 1 is subtract (eq. 4) and if T_{max} between 80°F – 87°F and RH_{min} greater than 85%, adjustment 2 (eq. 5) is added.

$$adj1 = \frac{13 - RH_{min}}{4} \times \sqrt{\frac{17 - ABS(T_{max} - 0.95)}{17}} \text{ (eq. 4)}$$

$$adj2 = \frac{RH_{min} - 85}{10} \times \frac{87 - T_{max}}{5} \text{ (eq. 5)}$$

We then use the quadratic relationship identified in previous work[14] between HI_{max} and $WBGT_{in}$ to convert HI_{max} values to an approximated indoor $WBGT_{max}$ (eq. 6).

$$WBGT\ (^{\circ}C) = -0.0034HI^2 + 0.96HI - 34\ (HI\ in\ ^{\circ}F) \text{ (eq. 6)}$$

Outdoor wet bulb globe temperature ($WBGT_{out}$) is a linear combination of wet bulb temperature (T_w), black globe temperature (T_g) and dry bulb temperature (T_d) (eq. 7), whereas indoor wet bulb globe temperature ($WBGT_{in}$) combines only T_w and T_g (eq. 8).[14] Both require in-situ field instruments to correctly measure, though several methods exist to approximate $WBGT_{out}$ from meteorological data.[17]

$$WBGT_{out} = 0.7\text{Tw} + 0.2\text{Tg} + 0.1\text{Ta} \text{ (eq. 7)}$$

$$WBGT_{in} = 0.7\text{Tw} + 0.3\text{Tg} \text{ (eq. 8)}$$

We recognize that the $WBGT_{max}$ approximation used in this analysis assumes fixed wind speeds (0.5 m s^{-1}) and neglects radiated heat of $WBGT_{out}$. But given that incarcerated Americans spend the preponderance of their time indoors and that most carceral facilities lack AC, $WBGT_{in}$ is appropriate to measure how humid heat exposure and changed across carceral facilities in the continental United States.

Further, WBGT thresholds are used by multiple organizations, including the International Standards Organization (ISO) and the US National Institute for Occupational Safety & Health (NIOSH), to identify occupational risks related to heat stress and it is widely used in environmental epidemiological research across a range of context to assess relationship between heat and human health. For example, evidence from epidemiological research in Qatar found strong correlation between cardiovascular mortality among Nepali migrant workers and elevated monthly average $WBGT_{max}$[12] and recent research has demonstrated both outdoor and indoor wet bulb globe temperature are robust when assessing associations between short-term temperature exposure and various kidney diseases in New York State.[13]

Secondary analyses

We include additional analyses by further carceral facility types in the Supplementary Information (Supplementary Figures 1 – 2). We also present results from Figures 1 and 2 with alternative thresholds of 26°C and 30°C (Supplementary Figures 3 - 6).

Validation of $WBGT_{max}$

The PRISM dataset is extensively validated and deriving HI_{max} from PRISM has been shown to be appropriate to use for environmental epidemiological research, including identifying warm days across the United States. Nonetheless, numerous gridded observational climate datasets exist, including reanalysis products like the European Centre for Medium-Range Weather Forecasts (ECWMF) ERA5.[20] Recent findings suggests that, when correlated against station observations, the accuracy of both PRISM and ERA5 derived WBGT may vary by climate zone in the United States.[21]

To illuminate the degree to which PRISM-derived WBGT used in this analysis compares to WBGT derived from ERA5 (available at 31km) and from ground stations, we estimated the correlation between average summer month (May – September) daily T_{max}, RH_{min}, and $WBGT_{max}$ for PRISM, ERA5, and HadISD (a quality-controlled subset of ISD), available from the Met Office Hadley Centre for Climate Science and Services.[22,23] We selected stations within the contiguous United States, and then applied further quality-control steps as enumerated in [24], plus an additional requirement that less than 10% of days be missing during 2015 to 2020.

Supplementary Figure 7 shows the month Person's R^2 correlation for T_{max}, RH_{min}, and

$WBGT_{max}$ and suggests that ERA5 and PRISM are well-correlated at the national level for all three metrics. ERA5 tends to be better correlated with ISD observations of $WBGT_{max}$ most months, but PRISM performs better in 2016 and 2020.

Because we are concerned with hot days, we reperform the analysis of Figure 1, but for only to include days ISD stations measured T_{max} greater than 26°C (Supplementary Figure 8). The correlation is weaker for PRISM for hotter days compared to ERA5, though not for all months. Both ERA5 and PRISM are widely used in environmental epidemiological research and both datasets have been expensively validated. The weaker correlation of PRISM we present here merits further investigation. As noted above, emerging research[21] suggests that the strength of the correlation depends on the climate region of the stations. But a full intercomparison it is outside the scope of this research brief.

Calculating humid heat exposure and trajectories of change metrics

For each carceral facility, we calculated the number of days in each year during 1982-2020 that were greater than 28°C $WBGT_{max}$ (n_days_{year}). We first assigned the average number of days per year $WBGT_{max}$ exceeded 28°C during 2016 - 2020. Then, we measured exposure during 2016 - 2020 by multiplying the number of incarcerated people housed at each carceral facility in 2018 by the average number of days $WBGT_{max}$ exceeded 28°C from 2016 – 2020:

$$\Sigma Temperature_{Person-Days} = n_{Days_{WBGT_{max}>28°C}} * Population \text{ (eq. 9)}$$

To calculate the disparities between carceral facilities with the rest of the state, we calculated state-level estimates for number of days over 28°C by aggregating across counties in each state in each year using county-level population weights derived from the NCHS Vintage 2020 bridged-race dataset during 1990 - 2019[25] and from the US Census Bureau prior to 1990:[26]

$$(n_{Days_{WBGT_{max}>28°C}})_{state} = \sum_{county}(n_{Days_{WBGT_{max}>28°C}})_{county} * Population\ Weight_{county} \text{ (eq. 10)}$$

We then made a population-weighted estimate of the state-level carceral facility value for estimates for number of days over 28°C, $(n_{Days_{WBGT_{max}>28°C}})_{carceral\ facilities}$, in a similar way to the state, and subtracted the estimate calculated for the entire state to obtain the annual estimated disparity in exposure to humid heat days in each year of study in each state:

$$Disparity_{state} = (n_{Days_{WBGT_{max}>28°C}})_{carceral\ facilities} - (n_{Days_{WBGT_{max}>28°C}})_{state} \text{ (eq. 11)}$$

To estimate trajectories of change in dangerous humid heat, we performed a linear regression of:

$$(n_{Days_{WBGT_{max}>28°C}})_{carceral\ facility} \sim \alpha_{carceral\ facility} + \beta_{carceral\ facility} * year \text{ (eq. 12)}$$

to estimate the annual change in $(n_{Days_{WBGT_{max}>28°C}})_{carceral\ facility}$ per year from 1982 – 2020, $\beta_{carceral\ facility}$. Using this fitted linear regression for each carceral facility, we then used the estimated parameter ($\beta_{carceral\ facility}$) multiplied by the number of years between 1982 - 2020 (37 years) to estimate the fitted change in number of humid heat days, $\Delta(n_{Days_{WBGT_{max}>28°C}})_{carceral\ facility}^{*}$:

$$\Delta(n_{Days_{WBGT_{max}>28°C}})_{carceral\ facility}^{*} = 37 * \beta_{carceral\ facility} \text{ (eq. 13)}$$

Incarcerated vs. non-incarcerated statistics

Black and Hispanic people are disproportionately represented within state prisons and local jails relative to the general U.S. population and to the state populations in California, Arizona, and Texas (Supplementary Tables 1 and 2). Compared to the general U.S. population, incarcerated people have higher rates of unemployment and homelessness (pre-incarceration), are less likely to have at least a high school education and are more likely to have an income of < $22,500 (pre-incarceration).

References

1. J. Skarha, *et al.*, Heat-related mortality in U.S. state and private prisons: A case-crossover analysis. *PLOS ONE* 18, e0281389 (2023).

2. D. Holt, Heat in US Prisons and Jails: Corrections and the Challenge of Climate Change, Sabin Center for Climate Change Law (2015) https:/doi.org/10.2139/ssrn.2667260

3. J. Skarha, M. Peterson, J. D. Rich, D. Dosa, An Overlooked Crisis: Extreme Temperature Exposures in Incarceration Settings. *Am. J. Public Health* 110, S41–S42 (2020).

4. A. R. Colucci, D. J. Vecellio, M. J. Allen, Thermal (In)equity and incarceration: A necessary nexus for geographers. *Environ. Plan. E Nat. Space* 6, 638–657 (2023).

5. Dogbeh, A., Jomaas, G., Bjarløv, S. P., & Toftum, J. (2015). Field study of the indoor environment in a Danish prison. Building and Environment, 88, 20-26.

6. Vant-Hull, B., Ramamurthy, P., Havlik, B., Jusino, C., Corbin-Mark, C., Schuerman, M., ... & Glenn, A. A. (2018). The Harlem heat project: A unique media–community collaboration to study indoor heat waves. Bulletin of the American Meteorological Society, 99(12), 2491-2506.

7. U.S. Department of Homeland Security, HIFLD Open Data, https://hifld-geoplatform.opendata.arcgis.com, Accessed July 10, 2023.

8. PRISM Climate Group, Oregon State University, https://prism.oregonstate.edu, data created 4 Feb 4, 2014, accessed Jul 10, 2023.

9. C. Daly, J. I. Smith, K. V. Olson, Mapping Atmospheric Moisture Climatologies across the Conterminous United States. *PLOS ONE* 10, e0141140 (2015).

10. Daly, C., Halbleib, M., Smith, J. I., Gibson, W. P., Doggett, M. K., Taylor, G. H., ... & Pasteris, P. P. (2008). Physiographically sensitive mapping of climatological temperature and precipitation across the conterminous United States. International Journal of Climatology: a Journal of the Royal Meteorological Society, 28(15), 2031-2064.

11. Spangler, K. R., Weinberger, K. R., & Wellenius, G. A. (2019). Suitability of gridded climate datasets for use in environmental epidemiology. Journal of exposure science & environmental epidemiology, 29(6), 777-789.

12. Pradhan, B., Kjellstrom, T., Atar, D., Sharma, P., Kayastha, B., Bhandari, G., & Pradhan, P. K. (2019). Heat stress impacts on cardiac mortality in Nepali migrant workers in Qatar. Cardiology, 143(1-2), 37-48.

13. Chu, L., Chen, K., Crowley, S., & Dubrow, R. (2023). Associations between short-term temperature exposure and kidney-related conditions in New York State: The influence of temperature metrics across four dimensions. Environment International, 173, 107783.

14. Bernard, T. E., & Iheanacho, I. (2015). Heat index and adjusted temperature as surrogates for wet bulb globe temperature to screen for occupational heat stress. Journal of

Occupational and Environmental Hygiene, 12(5), 323-333.

15. Tuholske, C., Caylor, K., Funk, C., Verdin, A., Sweeney, S., Grace, K., ... & Evans, T. (2021). Global urban population exposure to extreme heat. Proceedings of the National Academy of Sciences, 118(41), e2024792118.

16. National Weather Service, Heat Index Equation, https://www.wpc.ncep.noaa.gov/html/heatindex_equation.shtml Accesssed July 10, 2023).

17. Q. Kong, M. Huber, Explicit Calculations of Wet-Bulb Globe Temperature Compared With Approximations and Why It Matters for Labor Productivity. *Earths Future* 10, e2021EF002334 (2022).

18. Pradhan, B., Kjellstrom, T., Atar, D., Sharma, P., Kayastha, B., Bhandari, G., & Pradhan, P. K. (2019). Heat stress impacts on cardiac mortality in Nepali migrant workers in Qatar. Cardiology, 143(1-2), 37-48.

19. Chu, L., Chen, K., Crowley, S., & Dubrow, R. (2023). Associations between short-term temperature exposure and kidney-related conditions in New York State: The influence of temperature metrics across four dimensions. Environment International, 173, 107783.

20. Hersbach, H., Bell, B., Berrisford, P., Hirahara, S., Horányi, A., Muñoz-Sabater, J., ... & Thépaut, J. N. (2020). The ERA5 global reanalysis. Quarterly Journal of the Royal Meteorological Society, 146(730), 1999-2049.

21. Ahn, Y., Tuholske, C., & Parks, R. M. (2023). Comparing Approximated Heat Stress Measures Across the United States. https://doi.org/10.21203/rs.3.rs-3186416/v1

22. Dunn, R. J., Willett, K. M., Thorne, P. W., Woolley, E. V., Durre, I., Dai, A., ... & Vose, R. S. (2012). HadISD: A quality-controlled global synoptic report database for selected variables at long-term stations from 1973–2011. Climate of the Past, 8(5), 1649-1679.

23. Dunn, R. J., Willett, K. M., Parker, D. E., & Mitchell, L. (2016). Expanding HadISD: Quality-controlled, sub-daily station data from 1931. Geoscientific Instrumentation, Methods and Data Systems, 5(2), 473-491.

24. Raymond, C., Matthews, T., & Horton, R. M. (2020). The emergence of heat and humidity too severe for human tolerance. Science Advances, 6(19), eaaw1838.

25. U.S. Census Bureau, U.S. Census Populations With Bridged Race Categories (2022), Accessed, https://www.cdc.gov/nchs/nvss/bridged_race.htm Accessed July 17, 2023.

26. U.S. Census Bureau, U.S. Bureau, County Intercensal Tables 1980-1990. https://www.census.gov/data/tables/time-series/demo/popest/1980s-county.html Accessed July 17, 2023).

Supplementary Table 1. Race, sex, and some SES variables for state incarcerated populations compared to U.S. general population.

Variable	State incarcerated population	U.S. general population (18+)	Source
Race			Beyond the count: A deep dive into state prison populations from the Prison Policy Initiative, 2022. https://nicic.gov/weblink/beyond-count-deep-dive-state-prison-populations-2022<br><br>-data are from Bureau of Justice Statistics' 2016 Survey of Prison Inmates<br>-data are not regularly collected; most recent year available
White	32%	62%	
Black	34%	12%	
Hispanic	21%	17%	
Sex			
Men	93%	49%	
Women	7%	51%	
Unemployment rate (pre-incarceration)	14.8%	4.7%	
Percent homeless (pre-incarceration)	4.9%	0.2%	
Percent with less than high school education	White: 52%<br>Black: 68%<br>Hispanic: 69%	Overall: 12%	
Percent with annual income of < $22,500 (pre-incarceration)	57%	23%	Prisons of poverty: Uncovering the pre-incarceration incomes of the imprisoned, 2015 https://www.prisonpolicy.org/reports/income.html

Note: the Prison Policy Initiative Reports are all based on Bureau of Justice Statistics

Supplementary Table 2. Race of incarcerated population compared to state population for CA, TX, AZ.

State	State incarcerated population	State general population	Source	Year
**California**			California's Prison Population Fact Sheet from Public Policy Institute of California, 2017 https://www.ppic.org/publication/californias-prison-population/	2017
Black men	28.5%	5.6%		
Black women	25.9%	5.7%		
Incarceration rate for black men	4,236 / 100,000			
Incarceration rate for white men	422 / 100,000			
**Texas**			Texas state profile from Prison Policy Initiative, 2023 https://www.prisonpolicy.org/profiles/TX.html	2021
Black	33% prisons, 28% jails	12%		
White	34% prisons, 40% jails	41%		
Hispanic	33% prisons, 31% jails	40%		
**Arizona**			Incarceration trends in Arizona from Prison Policy Initiative, 2023 https://www.prisonpolicy.org/profiles/AZ.html	2021
Black	15% prisons, 16% jails	4%		
White	38% prisons, 55% jails	53%		
Hispanic	39% prisons, 20% jails	32%		
NA/AN	6% prisons, 8% jails	4%		

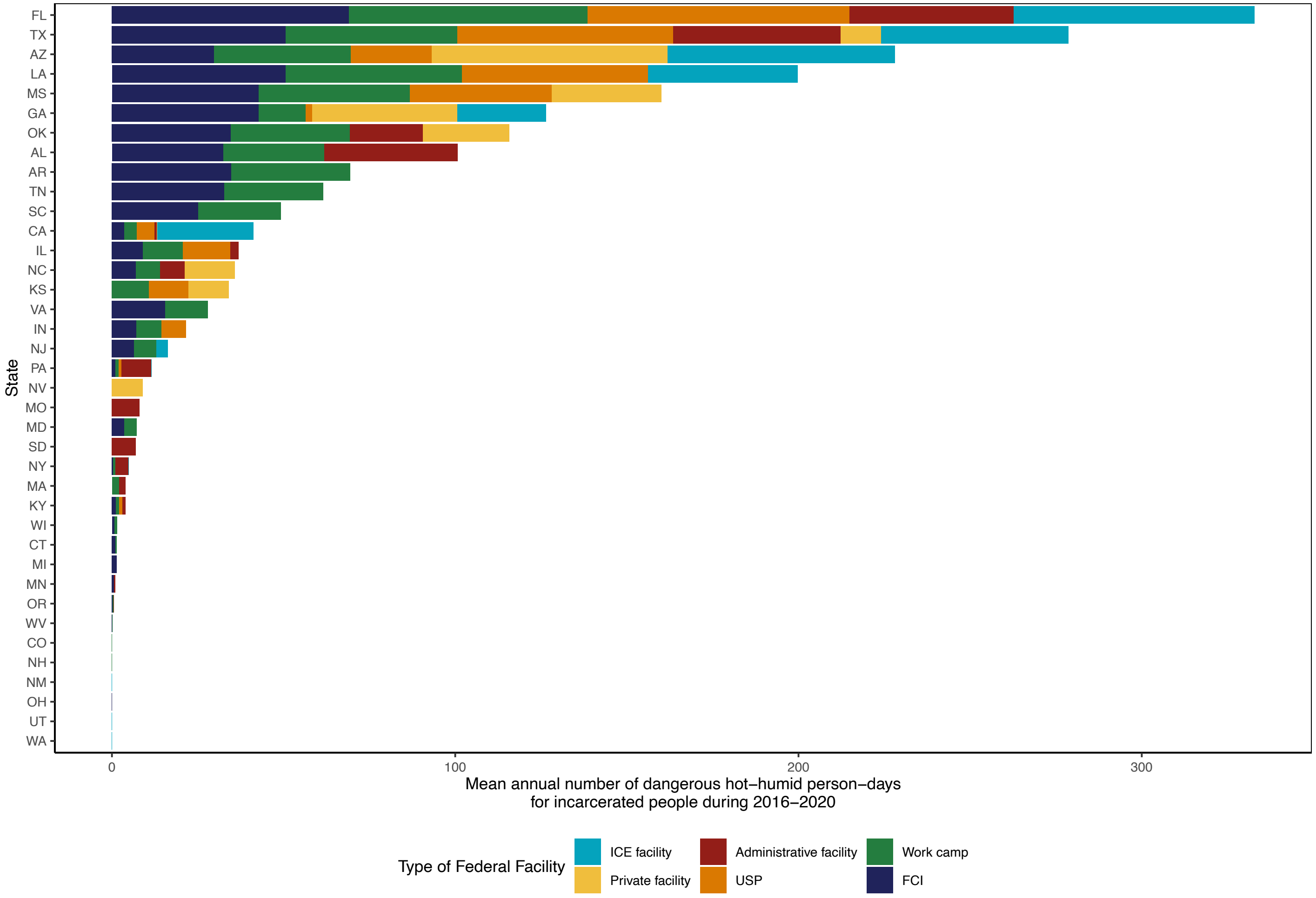

Supplementary Figure 1. Mean annual exposure during 2016 - 2020 to potentially hazardous heat in Federal carceral facilities within the continental United States (N = 232), measured by the number of person-days WBGTmax exceeded 28°C for incarcerated people by state and Federal facility type (Administrative, Federal Correctional Institution (FCI), Immigration and Customs Enforcement (ICE), Private, United States Penitentiary (USP), and Work camps).

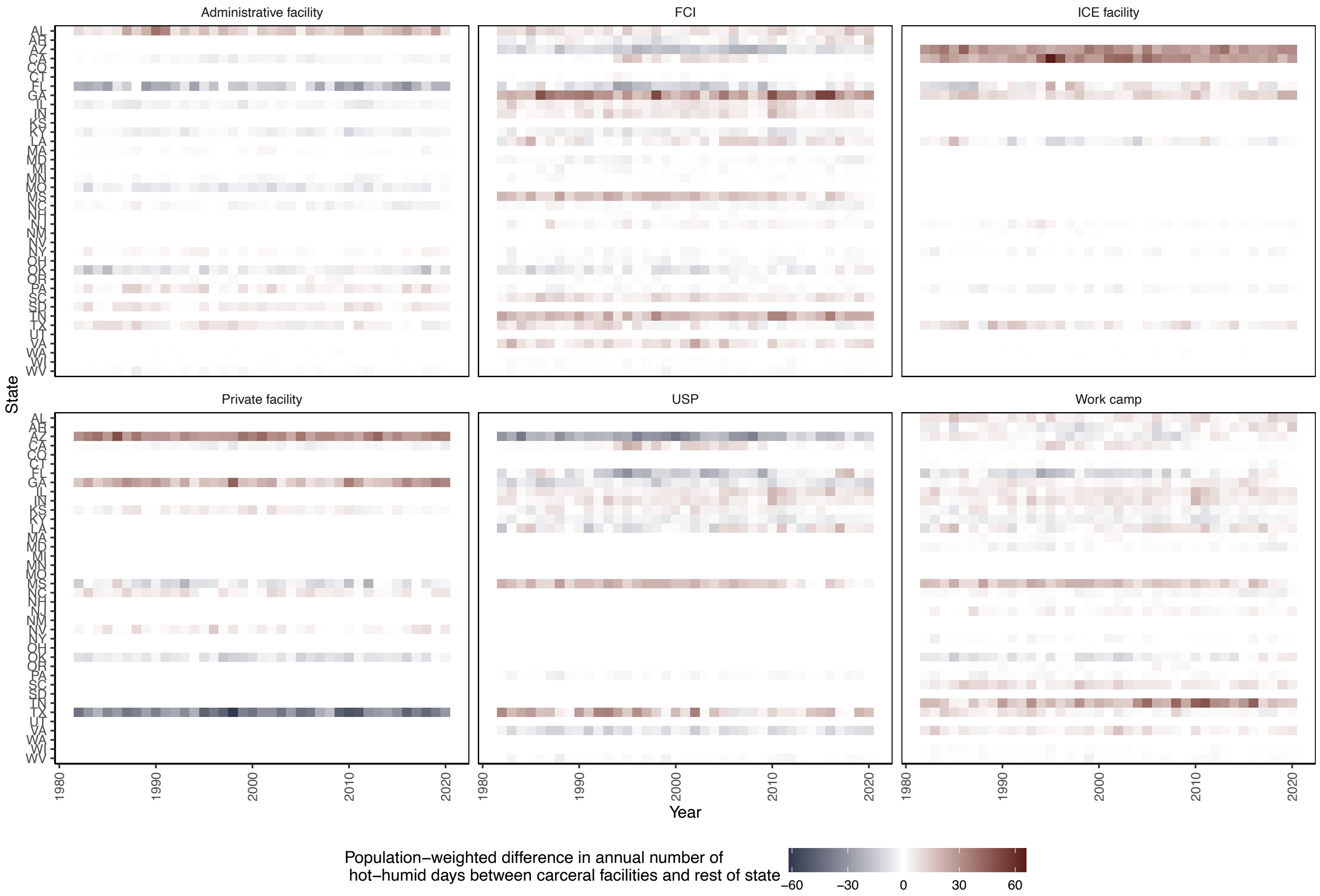

Supplementary Figure 2. The population-weighted difference between the annual number of days with heat stress conditions (defined as WBGTmax exceeding 28°C) at the location of Federal carceral facilities (Administrative, Federal Correctional Institution (FCI), Immigration and Customs Enforcement (ICE), Private, United States Penitentiary (USP), and Work camps) versus all other locations in the continental United States from 1982 - 2020 stratified by state.

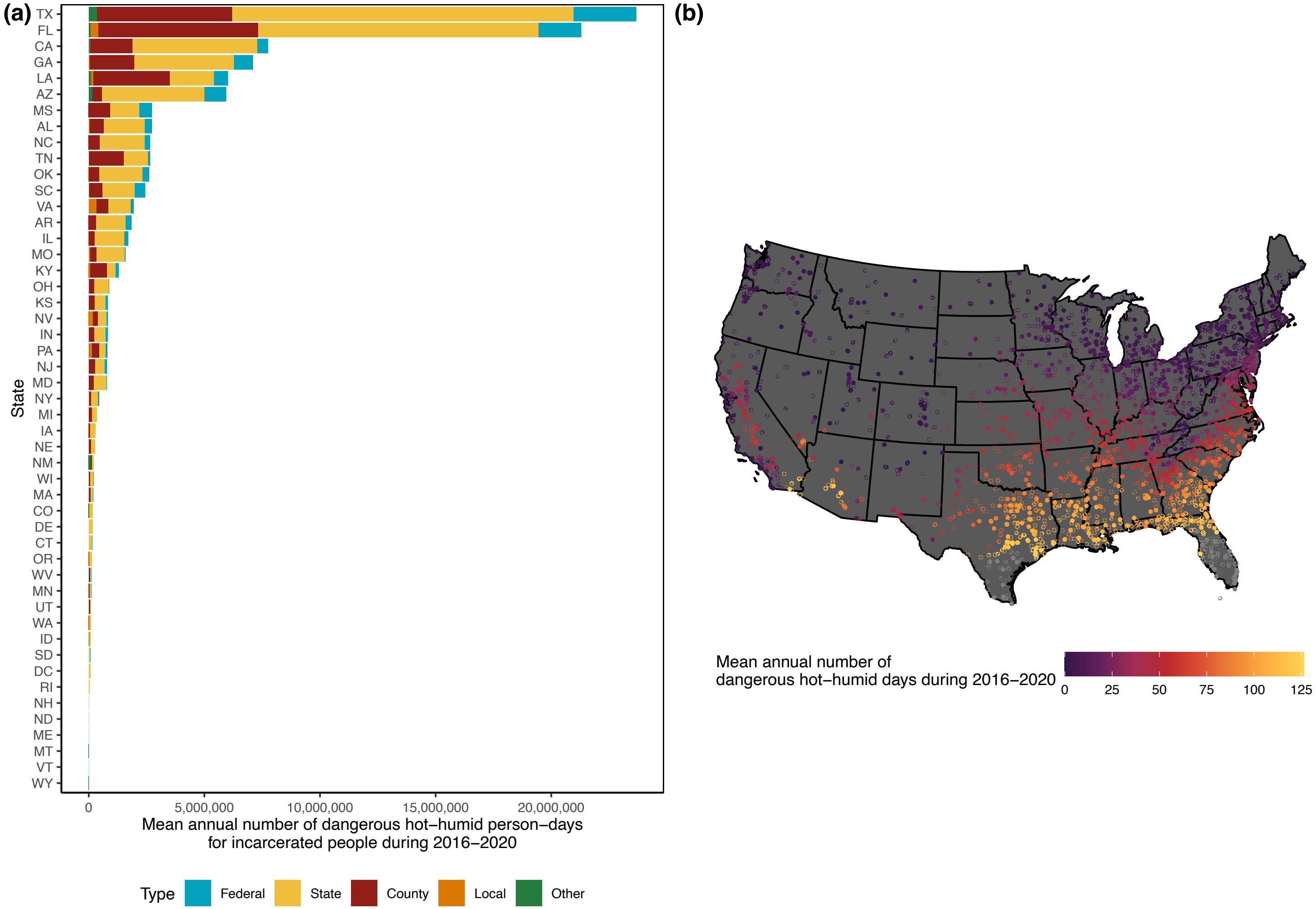

Supplementary Figure 3. Mean annual exposure during 2016 - 2020 to potentially hazardous heat in carceral facilities within the continental United States (N=4,078), measured by: (a) the number of person-days $WBGT_{max}$ exceeded 26°C for incarcerated people by state and carceral facility type; and (b) the number of days $WBGT_{max}$ exceeded 26°C for each carceral facility.

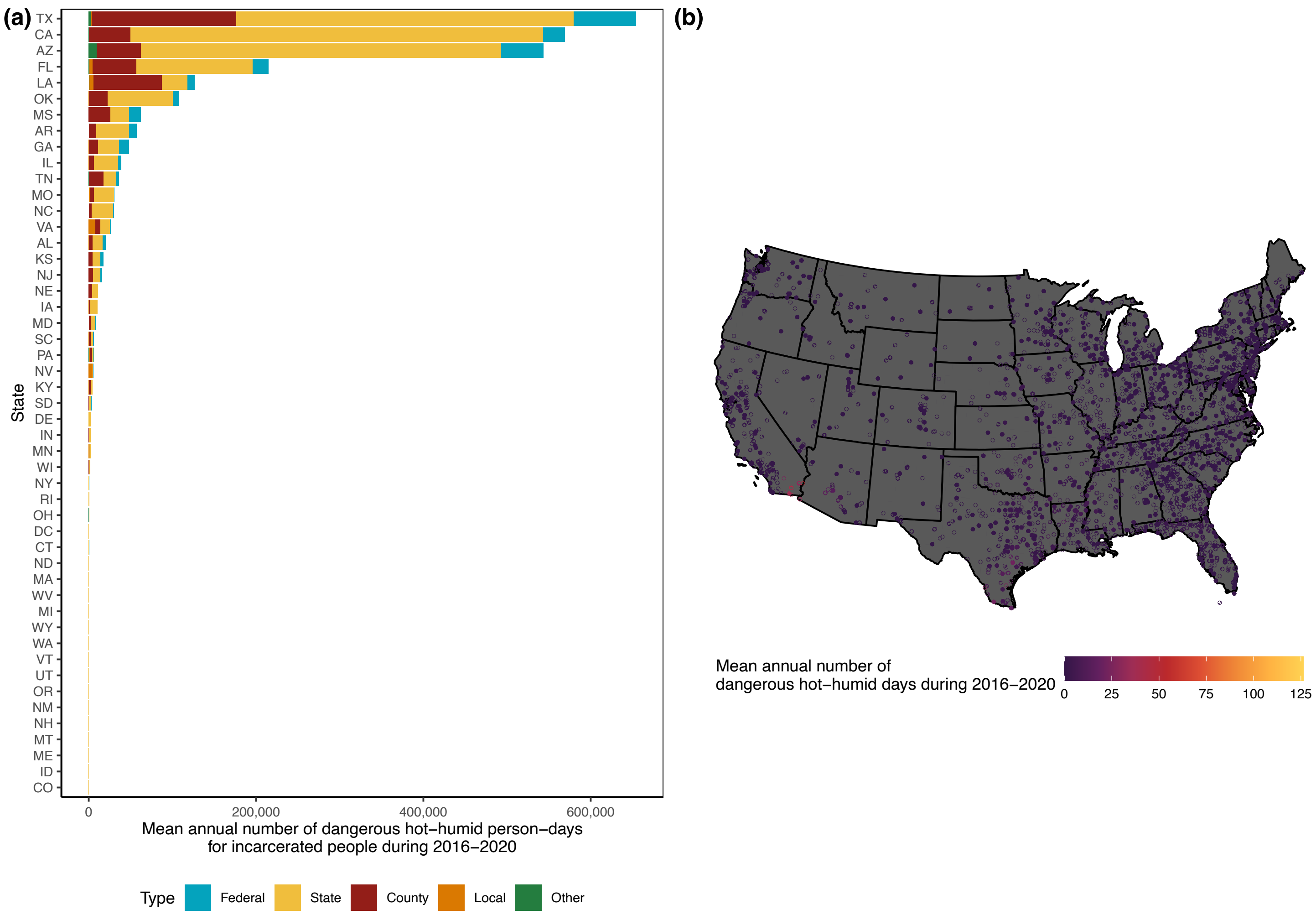

Supplementary Figure 4. Mean annual exposure during 2016 - 2020 to potentially hazardous heat in carceral facilities within the continental United States (N=4,078), measured by: (a) the number of person-days $WBGT_{max}$ exceeded 30°C for incarcerated people by state and carceral facility type; and (b) the number of days $WBGT_{max}$ exceeded 30°C for each carceral facility.

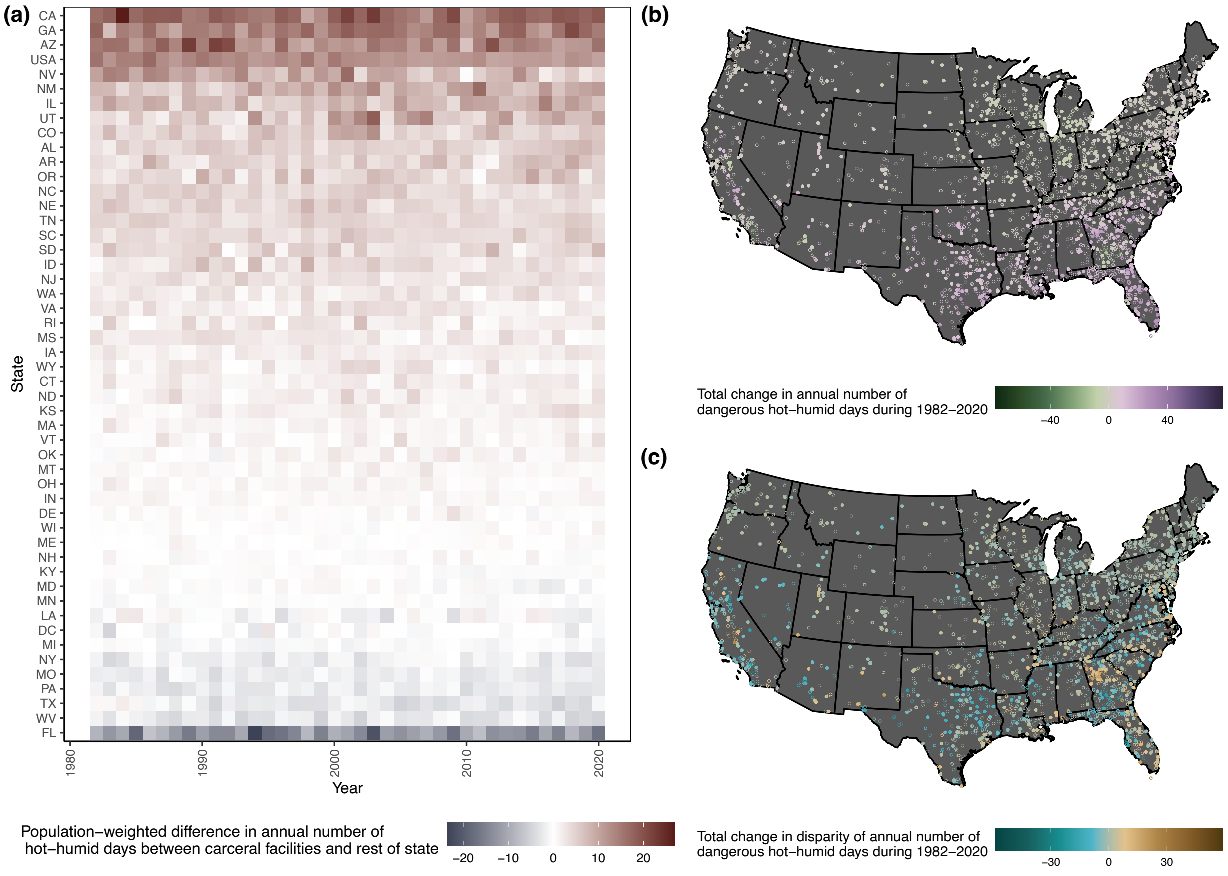

Supplementary Figure 5. (a) Population-weighted difference between the annual number of days $WBGT_{max}$ exceeded 26°C at the location of carceral facilities versus all other locations in the continental United States during 1982 – 2020, overall and stratified by state, ordered by average population-weighted difference, (b) the total change in the number of number of days $WBGT_{max}$ exceeded 26°C per year for each carceral facility in the continental United States during 1982 – 2020, and (c) the total change in disparity in number of number of days $WBGT_{max}$ exceeded 26°C per year for each carceral facility in the continental United States, compared with the rest of the state the carceral facility is located, during 1982 – 2020.

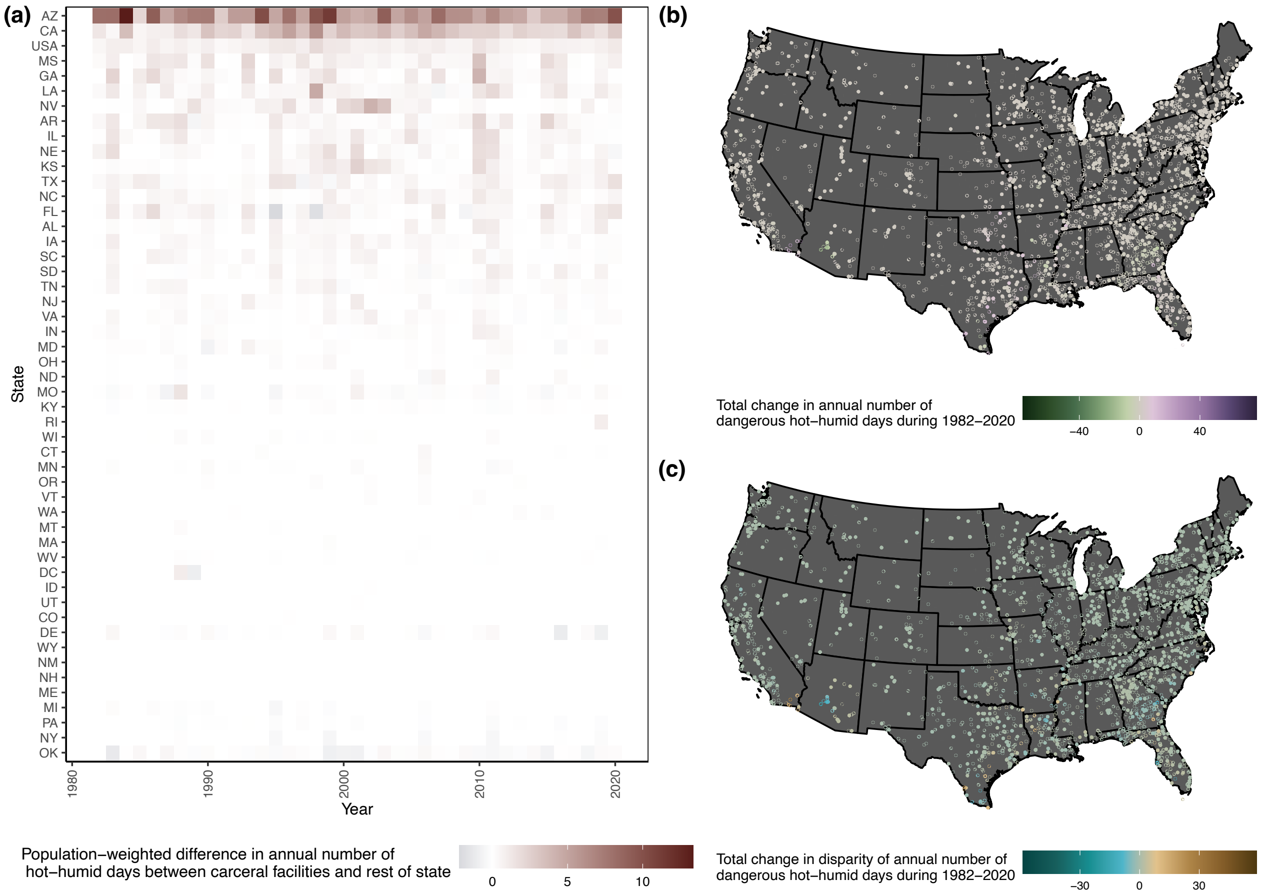

Supplementary Figure 6. (a) Population-weighted difference between the annual number of days $WBGT_{max}$ exceeded 30°C at the location of carceral facilities versus all other locations in the continental United States during 1982 – 2020, overall and stratified by state, ordered by average population-weighted difference, (b) the total change in the number of number of days $WBGT_{max}$ exceeded 30°C per year for each carceral facility in the continental United States during 1982 – 2020, and (c) the total change in disparity in number of number of days $WBGT_{max}$ exceeded 30°C per year for each carceral facility in the continental United States, compared with the rest of the state the carceral facility is located, during 1982 – 2020.

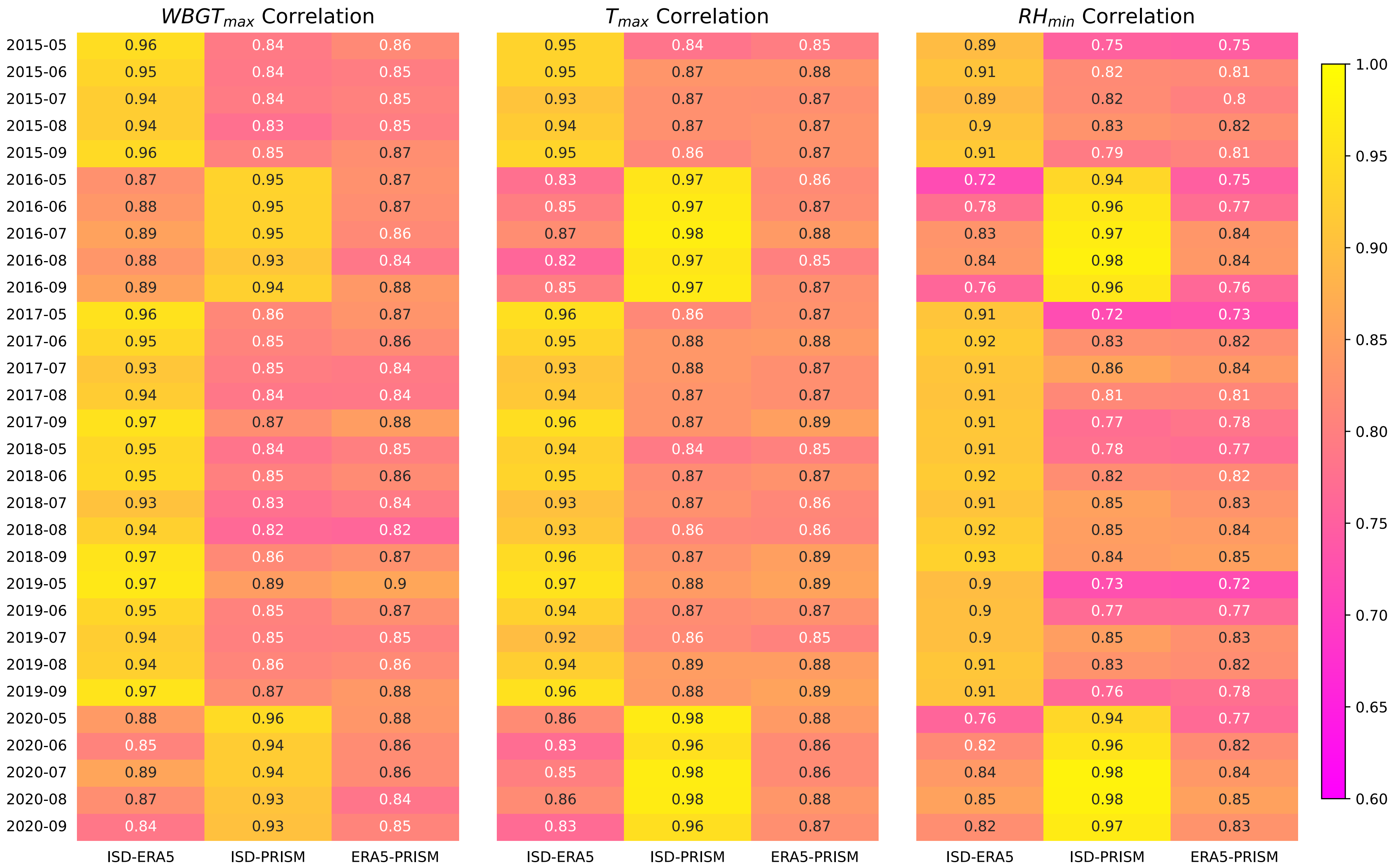

Supplementary Figure 7. Monthly correlation of daily T_{max}, RH_{min}, and $WBGT_{max}$ during 2015 – 2020 for HadISD stations, PRISM climate grids, and ERA5 reanalysis data for the United States.

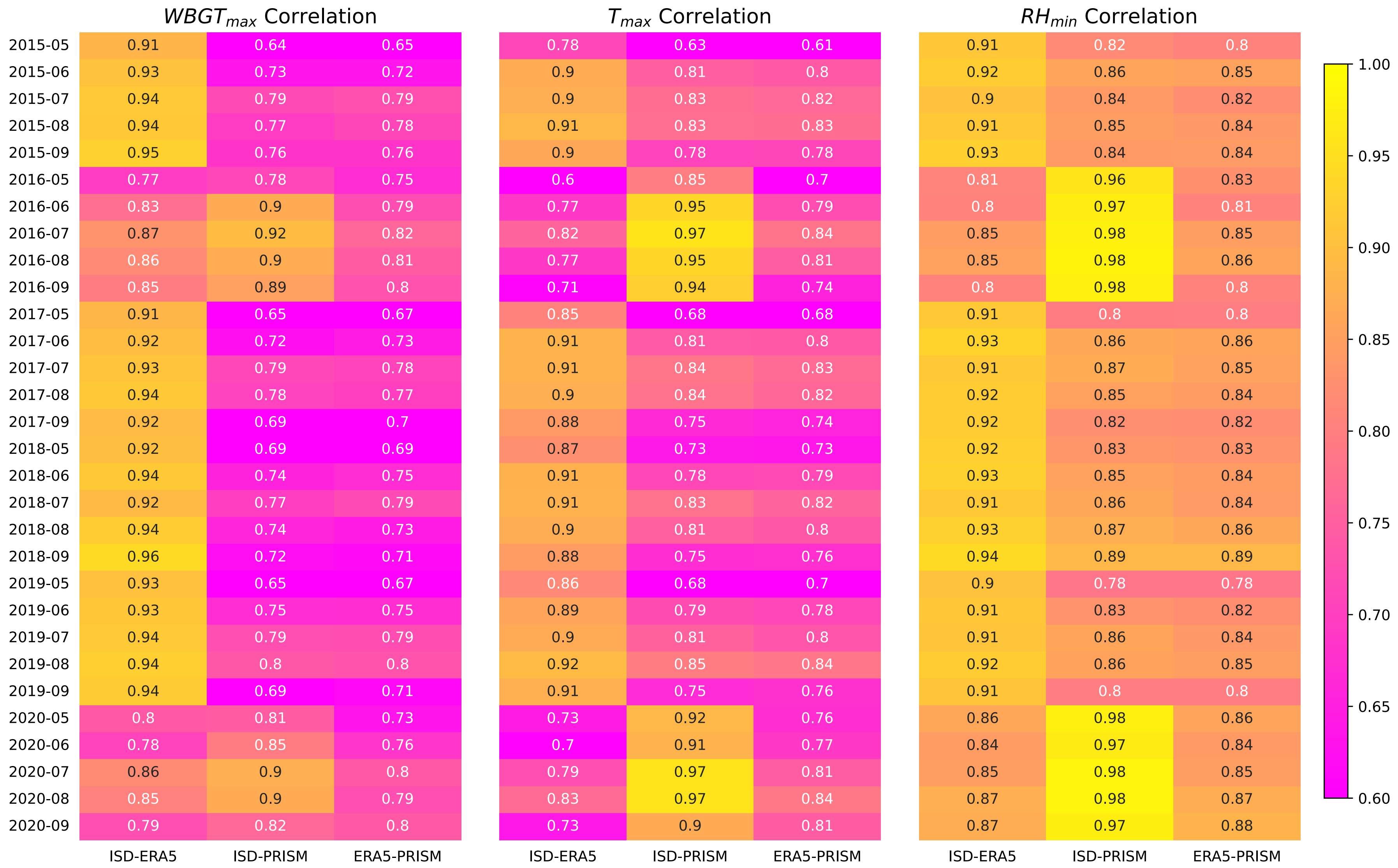

Supplementary Figure 8. Monthly correlation of daily T_{max}, RH_{min}, and $WBGT_{max}$ during 2015 – 2020 for HadISD stations, PRISM climate grids, and ERA5 reanalysis data for the United States for days HADISD stations reported T_{max} greater than 26°C.
